# Bioactives of *Momordica charantia* as Potential Anti-Diabetic/Hypoglycemic Agents

**DOI:** 10.3390/molecules27072175

**Published:** 2022-03-28

**Authors:** Bilin Xu, Zhiliang Li, Ting Zeng, Jianfeng Zhan, Shuzhen Wang, Chi-Tang Ho, Shiming Li

**Affiliations:** 1 College of Biology and Agricultural Resources, Huanggang Normal University, Huanggang 438000, China; xubilin@whu.edu.cn (B.X.); swlzl@hgnu.edu.cn (Z.L.); swzjf@hgnu.edu.cn (J.Z.); wangshuzhen710@whu.edu.cn (S.W.); 2 College of Pharmacy, Shandong University of Traditional Chinese Medicine, Jinan 250355, China; mm08901@yahoo.com; 3 Department of Food Science, Rutgers University, New Brunswick, NJ 08901, USA; ctho@sebs.rutgers.edu

**Keywords:** *Momordica charantia*, bioactives, hypoglycemic activity, diabetes mellitus, synergistic effect

## Abstract

*Momordica charantia* L., a member of the Curcubitaceae family, has traditionally been used as herbal medicine and as a vegetable. Functional ingredients of *M. charantia* play important roles in body health and human nutrition, which can be used directly or indirectly in treating or preventing hyperglycemia-related chronic diseases in humans. The hypoglycemic effects of *M. charantia* have been known for years. In this paper, the research progress of *M. charantia* phytobioactives and their hypoglycemic effects and related mechanisms, especially relating to diabetes mellitus, has been reviewed. Moreover, the clinical application of *M. charantia* in treating diabetes mellitus is also discussed, hoping to broaden the application of *M. charantia* as functional food.

## 1. Introduction

Due to globalization, industrialization and changes of human environment, hyperglycemia is widely prevalent [1]. Diabetes mellitus has been estimated to be the fifth leading cause of death globally, characterized by hyperglycemia due to defects in insulin action, insulin secretion, or both [2,3]. Characterized by altered carbohydrate, lipid and protein metabolism, diabetes mellitus is the leading cause of renal, neurological, and gastrointestinal manifestations in developed and developing countries [4,5,6,7]. Type 1 diabetes mellitus (T1DM), chiefly genetic, is characterized by disruption of pancreas function and absolute insulin insufficiency [8]. Insulin resistance plays a critical role in the development of type 2 diabetes mellitus (T2DM) and related complications [9]. T2DM is the major type of diabetes mellitus, affecting 90% of overall diabetes patients. Therefore, optimizing diabetes mellitus therapy is a modern critical medical and social challenge [8]. In particular, effective control of postprandial blood glucose levels may play key roles in diabetes care [10].

Many pharmacological approaches have been used to improve hyperglycemia, mainly through stimulating insulin release, increasing glucose transport activity, inhibiting gluconeogenesis, and reducing absorption of glucose from the intestine [10]. Currently, available therapies may be used as monotherapy or in combination to provide better glycemic regulation [11]. In addition to dietary management, hypoglycemic drugs are often used to treat T2DM [8]. However, the available hypoglycemic reagents have inadequate efficacy and some serious mechanism-based side effects, including hypoglycemia, gastrointestinal stimulation, and edema [12,13]. Owing to thesevere side-effects of synthetic hypoglycemic drugs, more effective and safer hypoglycemic agents from natural sources are greatly needed [14,15,16,17,18].

Natural phytoconstituents with hypoglycemic effect mainly contain peptides, lipids, glycopeptides, flavonoids, alkaloids, terpenoids, phenolics, glycosides, steroids, chalcones, carotenoids, tannins, saponins, iridoids, ursolic acid and imidazolines [19]. *Momordica charantia* L., also known as bitter melon or bitter gourd, belonging to the Cucurbitaceae family, is widely distributed in tropical and subtropical regions, such as Asia, South America, Africa, parts of the Amazon basin, and the Caribbean [20]. Notably, *M. charantia* seeds and fruits are rich in proteins, the quality of which meet the amino acids requirements/standards laid down by WHO for preschool children [21]. Due to various health-promoting properties, such as hypoglycemic, anti-cancerous, antimicrobial, antioxidant, antifertility, antimutagenic, antihelminthic, and immunomodulatory activities, *M. charantia* has been used as traditional medicinal plant in treating toothache, gout, jaundice, leprosy, furuncle, dysmenorrhea, piles, pneumonia, psoriasis, diarrhea, eczema, and rheumatism [22,23,24]. 

Due to remarkable hypoglycemic properties, *M. charantia* has great potential as an dietary ingredient and in medical foods for diabetic and prediabetic patients, as well as for the regulation of body weight and lipid metabolism [25]. This review discusses the potential applications of *M. charantia* bioactive compounds in the management of hyperglycemia and related chronic diseases, and clarifies the possible action of their mechanisms, hoping to supply valuable references for the development of *M. charantia* bioactives as hypoglycemic agents.

## 2. Bioactive Compounds of *M. charantia* with Hypoglycemic Potentials

**Figure 1:**
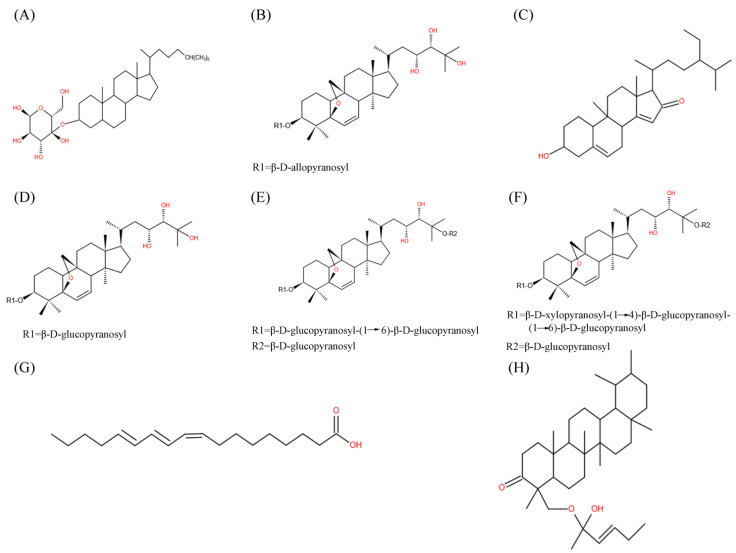
Chemical structure of main *M. charantia* active substance. (**A**–**H**) referred to charantin, karaviloside XI, momordenol, momordicoside Q, momordicoside S, momordicoside T, 9c,11t,13t-CLN, and momordicilin, respectively.

**Figure 2:**
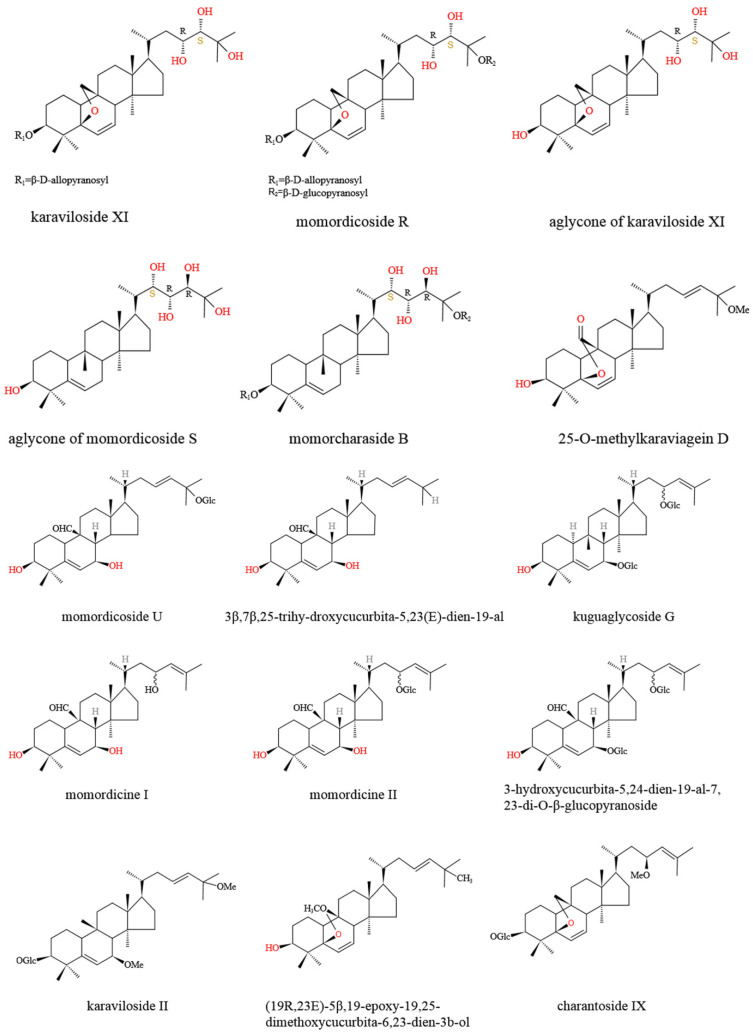
Chemical structure of main *M. charantia* cucurbitane-type triterpenoids.

As a highly nutritive vegetable, *M. charantia* contains numerous active phytochemicals, including proteins, carbohydrates, fatty acids, essential oils, amino acids, vitamins, phenolic acids, minerals, alkaloids, flavonoids, triterpenoids, quinines, saponins, triterpene glycosides, and other bioactive components [25]. Many types of bioactive compounds with hypoglycemic potentials have been isolated from *M. charantia*, among which cucurbitane-type triterpene glycosides, charantin, and momordicin were the well-studied compounds (Figure 1 and Figure 2). Polypeptide-p, purified from *M. charantia* fruit and seeds, showed effective hypoglycemic activities when administered subcutaneously to langurs, gerbils, and humans [26]. The 68-residue of insulin receptor (IR)-binding protein (mcIRBP) and adMc1proteins from *M. charantia* exhibited hypoglycemic effects in mice [27]. The 9cis,11trans,13trans-conjugated linolenic acid (9c,11t,13t-CLN) regulated lipid and glucose homeostasis via inducing acyl CoA oxidase (ACO) activity and serving as peroxisome proliferator activated receptor α (PPARα) activators [28]. Charantin, momordenol, and momordicilin are important active compounds possessing insulin-like chemical structure and properties [7]. Momordicine II and kuguaglycoside G could also stimulate insulin secretion [29,30].

*M. charantia* triterpenoids (Figure 1) were found to control the balance of blood glucose via increasing adenosine 5′-monophosphate (AMP)-activated protein kinase (AMPK) activity, which further enhanced glucose uptake and fatty acid oxidation, as well as inhibited lipid synthesis and hepatic glucose output [31]. Momordicosides (Q, R, S, U, and T) and karaviloside XI all exhibited many biologic effects beneficial to diabetes, such as enhancing the entry of inducible glucose into cells and stimulating fatty acid oxidation and glucose disposal [29,32,33]. The 5β,19-epoxy3β,25-dihydroxycucurbita- 6,23(E)-diene and 3β,7β,25-trihydroxycucurbita-5,23(E)-dien-19-al, purified from ether fraction of *M. charantia* methanol extract, showed blood hypoglycemic effects at the dosage of 400 mg/kg in diabetes-induced male ddY mice [33]. Protein tyrosine phosphatase 1B (PTP1B) served as a negative regulator of insulin through the dephosphorylation of the activated insulin receptor, which could be an effective target for the therapy of type 2 diabetes [34]. Cucurbitane-type triterpenoids, such as 25-O-methylkaraviagein D, karaviloside II, and (19R,23E)-5β,19-epoxy-19,25-dimethoxycucurbita-6,23-dien-3β–ol, all showed remarkable inhibitory activity against PTP1B and α-amylase (Figure 2) [15].

## 3. Antidiabetic Activity of *M. charantia*

*M. charantia* could effectively combat diabetes mellitus (Table 1). In obese diabetic *db/db* mice, *M. charantia* fruit water extract alone or together with platycodin-D significantly decreased obesity-related changes, and the water extract:platycodin-D (1:4) showed the most dramatic and synergic obesity-inhibiting effects [35]. In alloxan diabetic albino rats, acetone extract of whole *M. charantia* fruit lowered blood glucose from 13 to 50% after 8–30 days treatment [36]. Administration of *M. charantia* fruit methanol extract for 28 days lowered blood glucose levels in a dose-dependent manner in both normal and diabetic animals [37]. In the normal glucose primed rat model, alcoholic extract of *M. charantia* fruit significantly depressed plasma glucose levels by 10–15% at 1 h [38]. In both normal and streptozotocin-induced diabetic rats, subcutaneous administration of protein extract of *M. charantia* fruit pulp significantly decreased plasma glucose concentrations in a dose-dependent manner [39]. 

In normal rats, *M. charantia* pulp juice (250 mg/2 mL water) lowered fasting blood glucose levels significantly (*p* < 0.05 at 120 min), and the effect was even more pronounced with saponin-free methanol extract (150 mg/2 mL water) [40]. In diet-induced obesity C57BL/6 mice (male, 8 wk old), freeze-dried *M. charantia* fruit powder was seen to significantly reduce body weight, as the final body weights of mice receiving 10% *M. charantia* powder were almost the same as the control mice [41]. In normoglycemic Sprague-Dawley rats, aqueous extracts of *M. charantia* fruit (100 mg/kg) greatly reduced blood glucose levels [42].

In patients with T2DM, *M. charantia* extracts of unripe fruit could effectively lower the average fasting glucose level in an age- and sex-independent manner, showing no serious adverse events [43]. In a study of 52 individuals with prediabetes, *M. charantia* fruit extracts lowered elevated fasting plasma glucose [44]. Moreover, *M. charantia* fruit pulps at 2000 mg/day showed a modest hypoglycemic effect in patients with T2DM, and fructosamine levels were significantly reduced [45]. Among 112 patients with T2DM, administration of *M. charantia* fruit powder (2 or 4 g/day) significantly improved blood lipids, atherogenic index, body weight, and systolic blood pressure [46].

### 3.1. Improving Insulin Secretory and Resistance

*M. charantia* fruit extract could significantly increase islet size, number of β-cells, and total β-cell area, and also induce the regeneration of β-cells in the pancreatic islets of diabetic rats [47]. Furthermore, *M*. *charantia* fruit juice significantly increased the number of pancreatic β cells through reviving β cells and recovering partially destroyed β cells in streptozocin (STZ)-induced diabetic rats, but showed no effect on pancreatic α and δ cells [48]. In alloxan diabetic albino rats, acetone extract of *M. charantia* fruit showed antihyperglycemic activities through stimulating the recovery of pancreatic islet β cells [36]. Saponin-free methanol extract of *M. charantia* pulp juice (150 mg/2 mL water) showed significant hypoglycemic effects both in fasting (*p* < 0.05 at 120 min) and in postprandial states in non-insulin-dependent diabetes mellitus (NIDDM) model rats, through improving the insulin secretory capacity of B cells and enhancing insulin action, indicating the presence of non-sapogenin hypoglycemic compound(s) in *M. charantia* fruit pulp [40].

In MIN6 β-cells, *M. charantia* extracts rich in saponin significantly stimulated insulin secretion [29]. In particular, momordicine II and kuguaglycoside G also stimulated insulin secretion at concentrations of 10 μg/mL and 25 μg/mL, respectively [29]. The dried powder of *M. charantia* fruit pulp could also increase insulin secretion [49]. In INS-1 cells and rat pancreatic islets, *M. charantia* green fruit methanol extract and its ethyl acetate fraction could increase ATP content, augment insulin secretion in a dose-dependent manner, increase serum insulin levels after glucose loading, and decrease blood glucose levels significantly [50]. In MIN6 β-cells, purified momordicoside U (15.8–197.2 μM) moderately enhanced insulin secretion [30]. In both normal and streptozotocin-induced diabetic rats, protein extract of *M. charantia* fruit pulp raised plasma insulin concentrations 2-fold at 4 h following subcutaneous administration [39]. The protein extract of *M. charantia* fruit pulp (10 μg/mL) increased insulin secretion in perfused rat pancreases, and exerted insulin secretagogue and insulinomimetic activities to lower blood glucose concentrations [39]. In male high-fat-fed (HFD) Wistar rats, *M. charantia* fruit extract notably improved insulin sensitivity, and reduced fasting insulin [51].

**Figure 3:**
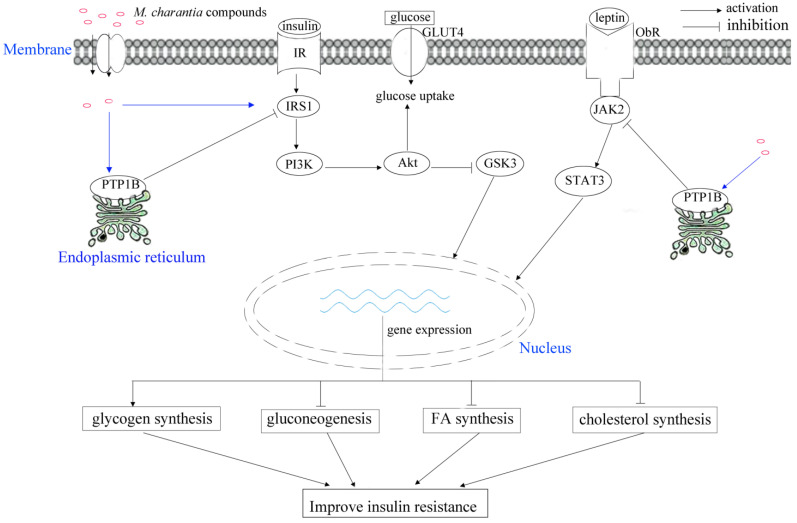
Schematic illustrating of *M. charantia* active substance towards diabetes mellitus.

The mcIRBP exhibited hypoglycemic effects in mice through interaction with insulin receptors (IR) [27]. In particular, the mcIRBP-19 (spanning residues 50–68 of mcIRBP) could enhance the binding of insulin to IR, stimulate phosphorylation of PDK1 and Akt, as well as stimulate the uptake of glucose in cells and clearance of glucose in diabetic mice (Figure 3) [52]. *M. charantia* fruit extract supplementation together with a high-fat diet (HFD) improved the insulin-stimulated tyrosine phosphorylation of insulin receptor subtrate-1 (IRS-1) [51]. In rats fed high fat diets, *M. charantia* freeze-dried unripe fruit juice (0.75%) could improve insulin resistance, as well as lower serum insulin and leptin [53]. 

**Table 1:** Effects of *M. charantia* active components on diabetes mellitus.

Active Components	Dose	Model	Effect	References
water extract: platycodin-D (1:4)	_	obese diabetic *db/db* mice	decrease obesity-related changes	[35]
acetone extract of whole fruit	25–75 mg/100 g body weight	alloxan diabetic albino rats,	lower blood glucose, stimulate the recovery of pancreatic islet β cells	[36]
methanol extract of fruit	200–600 mg/kg	normal and diabetic animals	lower blood glucose level,	[37]
alcoholic extract of fruit	500 mg/kg	normal glucose primed rat	depress plasma glucose levels, enhance glycogen synthesis in liver	[38]
protein extract of fruit pulp	5–10 mg/kg	normal and STZ-induced diabetic rats	exert insulin secretagogue and insulinomimetic activities, decrease plasma glucose concentrations, raise plasma insulin concentrations	[39]
fruit pulps	2000 mg/day	patients with T2DM	hypoglycemic effect	[45]
powder	2–4 g/day	patients with T2DM	improve blood lipids, atherogenic index, body weight, and systolic blood pressure,	[46]
fruit	_	STZ-induced diabetic rats, male high-fat-fed Wistar rats, rat L6 myotubes, male Sprague-Dawley rats with diabetes	increase number of pancreatic β cells, improve insulin sensitivity, reduce fasting insulin, increase glucose uptakes, improve wound healing, increase diversity and shift overall structure of gut microbiota, stimulate amino acid uptake, normalise structural abnormalities of peripheral nerves, reduce glucose absorptions, improve body mass gain and LDL cholesterol values	[48,51,54,55,56,57,58]
Saponin-free methanol extract of juice	150 mg/2 mL water	NIDDM model rats	improve insulin secretory capacity of B cells, enhance insulin action	[40]
saponin-rich fraction of fruit	125 μg/mL	MIN6 β-cells	stimulate insulin secretion	[29]
dried powder of fruit pulp	2000 mg/day	patients with T2DM	ameliorate diabetes associated CV risk, decrease level of glycosylated hemoglobin, increase insulin secretion	[46,49,59]
green fruit methanol extract and ethyl acetate fraction		INS-1 cells and rat pancreatic islets	increase ATP content, augment insulin secretion, increase serum insulin levels, decrease blood glucose levels	[50]
momordicoside U	15.8–197.2 μM	MIN6 β-cells	enhance insulin secretion	[30]
Momordicilin			block the active site of GSK-3,	[7]
mcIRBP			induce expression of GLUT4, stimulate phosphorylation of PDK1 and Akt, stimulate the uptake of glucose and clearance of glucose,	[27]
freeze-dried unripe fruit juice	0.75%	rats fed high fat diets	improve insulin resistance, lower serum insulin and leptin, improve oral glucose tolerance, lower body weight and visceral fat mass, raise serum-free fatty acid concentration, reduce adiposity,	[53]
momordicosides (Q, R, S, and T) and karaviloside XI	_	L6 myotubes, 3T3-L1 adipocytes, mice	enhance AMPK activity, stimulate GLUT4 translocation to the cell membrane, enhance fatty acid oxidation and glucose disposal	[32]
nanoparticles synthesized with filtrate of methanolic extract and silver nitrate	50 mg/kg	STZ-induced diabetic rats	regulate signaling pathways, up-regulate expression level of glucokinase	[60]

### 3.2. Regulating Glucose Uptake

Glucose transporters (GLUT) are widely distributed in body cells, facilitating the maintenance of the blood glucose level in the human body [61,62]. Sodium-coupled glucose transporters (SGLUTs) are scattered across the human body, and the selective inhibition of SGLUT1 could significantly slow postprandial gut uptake of glucose, as well as increase plasma levels of GLP-1 and GIP in healthy volunteers [63,64]. GLUT2 plays bidirectional roles in specific transportation of glucose in hepatocytes, as well as the absorption and reabsorption of glucose from enterocytes and renal tubules particularly [65]. Therefore, GLUT2 is considered as a competent target in treating diabetes mellitus [66]. The potential target proteins in diabetes contain dipeptidyl peptidase-IV (DPP-IV), GLUT, SGLTs, peroxisome proliferator-activated receptors, and α-glucosidase inhibitors [67]. The mcIRBP-19 could induce expression of GLUT4 (Figure 3) [52].

**Figure 4:**
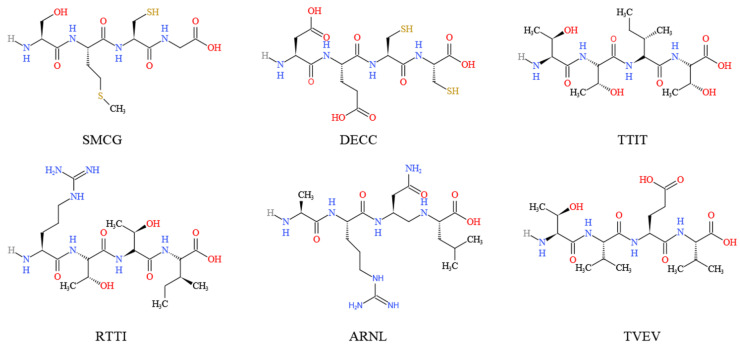
Chemical structure of the main *M. charantia* peptides derived from the hypoglycemic protein adMc1.

Six peptides (i.e., SMCG, DECC, TTIT, RTTI, ARNL and TVEV) (Figure 4), derived from the hypoglycemic protein adMc1 of *M. charantia*, were shown to be potential inhibitors of DPP-IV, SGLT1, and GLUT2 receptor proteins [66]. In L6 myotubes and 3T3-L1 adipocytes, momordicosides (Q, R, S, and T) and karaviloside XI could enhance AMPK activity and stimulate GLUT4 translocation to the cell membrane, which is an essential step for inducible glucose entry into cells [32]. In STZ-induced diabetic rats, daily oral administration of *M. charantia* fruit juice significantly reduced the Na^+^- and K^+^-dependent absorptions of glucose by jejunum [54]. Also in STZ-induced diabetic rats, *M. charantia* fruit juice could, like insulin, regulate glucose uptake into the jejunum membrane brush border vesicles and skeletal muscle cells [54]. In rat L6 myotubes, lyophilized extract of *M. charantia* fruit juice (5 µg/mL) stimulated the uptake of ^14^C-D-glucose, but high concentrations (10–200 µg/mL) inhibited the uptake [54]. *M. charantia* fruit protein extract enhanced glucose uptake into C_2_C_12_ myocytes and 3T3-L1 adipocytes, and significantly increased glucose uptake after 4–6 h of incubation in rat adipocytes [39]. Incubation of L6 rat myotubes with *M. charantia* fruit juice (1, 5 and 10 µg/mL) resulted in time-dependent increases in ^3^H-deoxy-D-glucose uptakes [55]. 

### 3.3. Improving Glucose Metabolism

Diabetes mellitus is associated with irregular glucose homeostasis, so the effective control of blood glucose level is critical in preventing or reversing diabetic complications and improving life quality in diabetic patients [68]. In male HFD Wistar rats, *M. charantia* fruit extract notably improved glucose tolerance [51]. In STZ-induced diabetic rats, alcoholic extract of *M. charantia* fruit improved the oral glucose tolerance, and led to significant reduction in plasma glucose of 26% at 3.5 h [38]. In rats fed high-fat diets, *M. charantia* freeze-dried unripe fruit juice (0.75%) could improve oral glucose tolerance [53]. The hypoglycemic activities of *M. charantia* fruit extracts might partly be due to increased glucose utilization in the liver [38]. *M. charantia* leaf nanoparticles, synthesized with filtrate from methanolic extract with silver nitrate (1 mM), could significantly up-regulate the expression level of glucokinase in diabetic rats [60]. Momordicilin exhibited antidiabetic activities through blocking the active site of glycogen synthase kinase-3 (GSK-3), which can phosphorylate and inactivate glycogen synthase [7]. In normally fed rats, alcoholic extract of *M. charantia* fruit (500 mg/kg) enhanced glycogen synthesis (4–5 fold) from U-^14^C-glucose in the liver [38].

### 3.4. Modulating Lipid and Amino Acid Metabolism

In male HFD Wistar rats, *M. charantia* fruit extract notably reduced triacylglycerol, cholesterol and epidydimal fat [51]. Momordicoside(s) could enhance fatty acid oxidation and glucose disposal in both insulin-sensitive and insulin-resistant mice [32]. In STZ-induced diabetic rats, *M. charantia* fruit powder (10 or 50 g/kg diet for 6 weeks) could improve body mass gain and low-density lipoprotein (LDL) cholesterol values, which could be dampened by co-administered trivalent chromium (Cr) [56]. In rats fed high-fat diets, *M. charantia* freeze-dried unripe fruit juice (0.75%) could lower body weight and visceral fat mass, raise serum free fatty acid concentration, and reduce adiposity without affecting fat absorption [53]. 

Microbes inhabiting the gut may play important roles in hosts’ metabolism homeostasis and health maintenance [69,70,71]. Oral administration of *M. charantia* fruit significantly prevented hyperlipidemia, but the effects substantially diminished when co-treated with antibiotics [57]. In particular, *M. charantia* fruit moderately increased diversity and shifted the overall structure of gut microbiota via enhancing the relative abundance of short-chain fatty acid (SCFAs)-producing genera and increasing fecal SCFAs content [57]. The transplantation of gut flora from *M. charantia* fruit-treated donor mice significantly decreased serum lipids in male recipient mice [57]. In L6 rat myotubes, *M. charantia* fruit juice (1, 5 and 10 µg/mL) enhanced the *N*-methyl-amino-α-isobutyric acid uptakes in a time-dependent manner, as *M. charantia* fruit juice exerted a hypoglycemic effect partly through stimulating amino acid uptake into skeletal muscle cells like insulin [55]. 

### 3.5. Protective Effects of M. charantia

In STZ-induced diabetic rats, *M. charantia* fruit juice normalized the structural abnormalities of peripheral nerves, including the mean cross-sectional myelinated nerve fibers, axonal area, myelin area, and maximal fiber area [54]. In Cr-co-supplemented type 2 diabetic rats, *M. charantia* fruit powder could decrease Cr content in liver and kidneys through binding of Cr by polyphenol-type compounds [55,56]. In STZ-induced diabetic rats, *M. charantia* leaf nanoparticles (50 mg/kg) could alleviate diabetes nephropathy through regulating SOCS/JAK/STAT and PI3K/Akt/PTEN signaling pathways: levels of Akt, PI3k, TGF-β, JAK2, STAT3 were down-regulated; the expressions of PTEN, SOCS3 and SOCS4 were up-regulated [60]. In type 2 diabetic *db/db* mice, the gastro-resistant peptide mcIRBP-9 showed anti-inflammatory and reno-protective abilities, as well as controlling blood glucose and HbA1c levels [72]. The mcIRBP-9 could ameliorate diabetic nephropathy through reducing renal vascular leakage and histopathological changes, altering pathways involved in inflammatory and immune responses, as well as improving inflammatory characteristics of mice [72]. In particular, nuclear factor-κB (NF-κB) played an important role in regulating mcIRBP-9-affected immune pathways [72]. In obese and diabetic OLETF rats, treatment with *M. charantia* edible portion (3%) down-regulated the levels of proinflammatory cytokines in liver, muscle and epididymal fats [73]. Administration of dried powder of *M. charantia* fruit pulp (2000 mg/day) significantly reduced the levels of glycated hemoglobin A1c, 2-h glucose, areas under the curve (AUC) of glucose, weight, fat percentage, body mass index, and waist circumference [49]. In 112 patients with T2DM, *M. charantia* fruit powder ameliorated diabetes-associated cardiovascular risk factors more effectively than glibenclamide [46]. In 40 diabetic patients (over 18 years old), *M. charantia* administration (two capsules, three times a day after meals, for 3 months) slightly decreased levels of glycosylated hemoglobin (hemoglobin A1c or HbA1c) by 0.22% [59]. *M. charantia* active components could reduce oxidative stress, decrease insulin resistance, increase insulin release, reduce adiposity, modulate glycolysis and gluconeogenesis, as well as lower oxidative status [74].

Diabetic patients often suffer from chronic nonhealing wounds, such as foot ulcers, which often result in amputations [75,76,77]. In diabetic patients, hyperglycemia can cause arteries to narrow, result in poor oxygenation of wound tissue, and delay wound repair and regeneration [58,78]. Moreover, the wound-healing response can be further compromised by chronic hyperglycemia-induced damage to both the peripheral nerves and the immune system [58,79]. Diabetes also has deleterious effects on granulation tissue cells, especially fibroblasts and endothelial cells [80]. In male Sprague-Dawley rats with diabetes, *M. charantia* fruit appeared to benefit the formation of wound granulation tissue, as distinct cellular layers were well-formed [75]. Moreover, *M. charantia* fruit treatment increased angiogenesis in diabetic granulation tissue, which was marked by abundant microvessels and large blood vessels [58]. In particular, locally applied *M. charantia* fruit extract could prevent regression of granulation tissue and blood vessels, and improve wound healing in diabetic wounds, showing no effect on systemic blood glucose levels or insulin receptor substrate 1 [58].

### 3.6. Inhibitory Effects of Related Enzymes

The α-glucosidase, located in the brush-border membranes of human intestinal cells, is involved in carbohydrate metabolism and the post-translational processing of glycoproptein [81]. The α-amylase, an important secretory product generated by the pancreas and salivary glands, can catalyze the initial step of starch hydrolysis to a mixture of oligosaccharides through cleavaging the α-D (1–4) glycosidic bonds [82,83]. In particular, α-glucosidase and α-amylase have long been proposed as candidate drug targets for the modulation of postprandial hyperglycemia [84].

**Figure 5:**
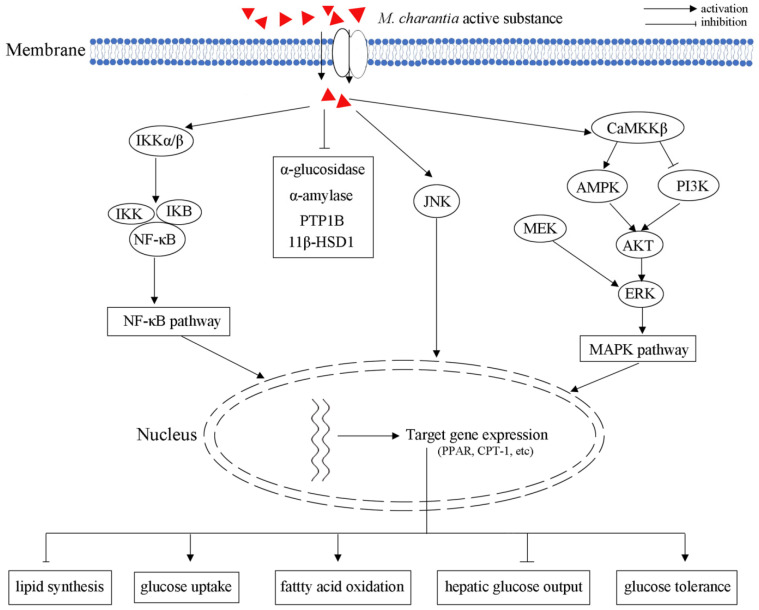
Schematic illustrating of *M. charantia* active substance towards obesity.

As natural inhibitors of α-glucosidase and α-amylase, *M. charantia* may be used as auxiliary hypoglycemic functional foods or drugs (Figure 5). Three cucurbitane-type triterpenoids, including 25-*O*-methylkaraviagein D, karaviloside II, and (19R,23E)-5β, 19-epoxy-19,25-dimethoxycucurbita-6,23-dien-3β–ol, could all inhibit α-glucosidase activity [15]. 25-*O*-Methylkaraviagein D showed remarkable inhibitory activity against PTP1B and α-amylase [15]. Capsules containing *M. charantia* extract also exerted anti-obesity activities through selectively and dose-dependently inhibiting the activity of 11β-Hydroxysteroid dehydrogenase type 1 (11β-HSD1), which is a microsomal enzyme converting glucocorticoid receptor-inert cortisone to active cortisol in metabolic tissues [85].

### 3.7. Regulation of Signal Pathways

AMPK plays multiple critical roles in the body’s overall metabolic balance, response to exercise, hormonal stimulation, nutritional stress, as well as glucose-lowering drugs metformin and rosiglitazone [10,86]. AMPK consists of a catalytic α subunit and two non-catalytic subunits (β and γ), forming active 1:1:1 heterotrimers. Moreover, the activation of AMPK can induce the expression of PPARα and carnitine palmitoyltransferase I (CPT-1), which further increase fatty acid oxidation and improve insulin sensitivity [87]. In L6 myotubes and LKB1-deficient HeLa cells, *M. charantia* triterpenoids increased AMPK activity by 20–35% through regulating the upstream kinase CaMKKβ in a Ca^2+^-independent manner [31]. As an AMPK activator, *M. charantia* triterpenoids could increase the expression of AMPK, and further control the balance of blood glucose (Figure 5).

PPARs could be activated by a ligand, heterodimerize with retinoid X receptor, binding to a peroxisome proliferator responsive element (PPRE), and promote transcription of target genes participating in lipid catabolism. Therefore, PPARs play important roles in regulating lipid and glucose homeostasis through genomic action [88]. The 9c,11t,13t-CLN, isolated from wild *M. charantia* fruit, could significantly induce ACO activity in a peroxisome proliferator-responsive murine hepatoma cell line (H4IIEC3) [28]. As a PPARα activator, 9c,11t,13t-CLN regulated lipid and glucose homeostasis through PPARα signaling pathways in vivo [28]. In obese and diabetic OLETF rats, treatment with *M. charantia* edible portion (3%) significantly improved glucose tolerance and insulin sensitivity via inhibiting NF-κB and JNK pathways: the levels of phospho-insulin receptor substrate-1 (Tyr612) and phospho-Akt (Ser473) were increased; the activation of NF-κB in liver and muscle was decreased [73].

## 4. Challenges and Perspectives

*M. charantia* has received considerable attention in biological and biomedical research due to its remarkable biological activities, especially its antidiabetic/hypoglycemic effects. *M. charantia* is usually served in one of four dosage forms (fruit juice, entire fruit, freeze-dried powder, or capsule), the preparations are mainly crude extracts (extracted with water, ethanol, or methanol) and the effective monomer components are extracted from fruit, seeds, and leaves [89]. In particular, the typical hypoglycemic activities are mainly attributed to proteins/peptides, polysaccharides, phenolic compounds, triterpenoids, alkaloids, and charantins [90,91]. As one of the most important global health problems, diabetes mellitus could be treated with several *M. charantia*-derived bioactive compounds, mainly through inhibiting α-glucosidase and α-amylase, activating AMPK, JNK, and Akt signal pathways, activating PTP1B activities, and inhibiting the formation of advanced glycation end-products (AGE). The modulation of gut microbiota is essential for the hypoglycemic and anti-hyperlipidemic activities of *M. charantia*. *M. charantia* fruit juice has multiple influences on glucose and lipid metabolism, strongly counteracting the untoward effects of high-fat diets. Furthermore, antioxidant and anti-inflammatory activities also greatly contribute to its anti-hyperglycemic properties. Long-term oral administration of *M. charantia* fruit extracts at appropriate dosages may be benefit in improving diabetes. Identification of potential mechanism(s) by which *M. charantia* improves insulin sensitivity and insulin signaling may supply new therapeutic targets for the treatment of obesity/dyslipidemia-induced insulin resistance.

Traditional *M. charantia* remedies have supplied sources of useful hypoglycemic agents, but should continue to be investigated for possible drug alternatives. In vitro and animal studies have suggested the remarkable hypoglycemic activity of *M. charantia*, but limited human research is available to support its usage. *M. charantia* has traditionally been used for treating diabetes, but some clinical trials show conflicting results. In addition, very limited-quality evidence has shown that *M. charantia* adjunct preparations could improve glycemic control in T2DM patients [92]. Moreover, no large clinical trial has been performed on the efficacy and safety of *M. charantia* preparation. Therefore, rigorous research focusing on standardizing *M. charantia* formulation is greatly needed, as well as clinical trials with adequate sample size to determine its efficacy and safety. Diabetes mellitus is also associated with an increase in sialic acid concentration, but ingestion of *M. charantia* fruit juice (55 mL/24 h) showed no effect on levels of serum sialic acid in NIDDM patients [93,94,95]. Moreover, diabetes mellitus is also associated with disruption of biorhythms, but no related research was available for active components of *M. charantia* [8]. Dysfunction of bone marrow-derived endothelial progenitor cells contributes to poor vasculogenesis in diabetes mellitus [96]. However, the effect of *M. charnatia* active components on bone marrow-derived endothelial progenitor cells is not well understood and needs to be studied in more depth. Moreover, the effect of *M. charantia* active components on protein kinase C is not well understood, and is vital when considering diabetic vascular complications [97].

Due to the interaction of drugs with in vivo systems, rational drug use should consider medical, biological, and pharmaceutical factors to ensure high bioavailability and efficacy. In particular, the most active candidates against diabetes mellitus will be determined through measuring many biochemical parameters, including fasting blood glucose, lipid profile, insulin, glycosylated hemoglobin, serum urea and creatinine, plasma alanine and aspartate transaminases, as well as microscopical examinations of pancreatic sections. Moreover, the gastrointestinal resistance of *M. charantia* bioactive compounds and their thermal tolerance in vivo also need to be better understood. Therefore, further study is greatly needed to investigate the detailed hypoglycemic mechanism and possible linkage to unexpected side effects, aiming to establish a safety guideline for the consumption of *M. charantia*-derived products.

## Data Availability

Not applicable.

## Abbreviations

AGE	advanced glycation end products
AMPK	Adenosine 5′-monophosphate (AMP)-activated protein kinase
adMc1	anti-hyperglycemic protein of *M. charantia*
AUC	areas under the curve
9c,11t,13t-CLN	9cis,11trans,13trans-conjugated linolenic acid
CPT-1	carnitine palmitoyltransferase I
Cr	chromium
DPP-IV	dipeptidyl peptidase-IV
GLUT	Glucose transporters
GLP-1	glucagon-like peptide 1
GIP	glucose-dependent insulinotropic peptide
HFD	high-fat-fed
IDDM	insulin-dependent diabetes mellitus
IR	insulin receptor
IRS-1	insulin receptor subtrate-1
NF-κB	nuclear factor-κB
NIDDM	non-insulin-dependent diabetes mellitus
PDK1	protein kinase-1
STZ	streptozocin
T1DM	Type 1 diabetes mellitus
T2DM	Type 2 diabetes mellitus
mcIRBP	*M. charantia* insulin receptor (IR)-binding protein
PPARα	Peroxisome proliferator activated receptor α
PTP1B	protein tyrosine phosphatase 1B
PPRE	peroxisome proliferator responsive element
SCFAs	short-chain fatty acid
SGLUTs	Sodium-coupled glucose transporters
SGLT1	sodium dependent glucose transporter 1
TAG	triacylglycerol
